# Community Pharmacists’ Practice, Awareness, and Beliefs about Drug Disposal in Saudi Arabia

**DOI:** 10.3390/healthcare9070823

**Published:** 2021-06-29

**Authors:** Sultan Alghadeer, Mohammed N. Al-Arifi

**Affiliations:** 1 Department of Clinical Pharmacy, College of Pharmacy, King Saud University, Riyadh 11451, Saudi Arabia; malarifi@ksu.edu.sa; 2 Research Center, Basic Sciences Department, Prince Sultan College for EMS, King Saud University, Riyadh 11451, Saudi Arabia

**Keywords:** community pharmacy, drug disposal, unused medication, environment, awareness, practice

## Abstract

The awareness among Saudi people regarding the good and safe practice of drug disposal is fairly low. Community pharmacists’ potential toward drugs disposal directions and practice are not emphasized enough. Therefore, a cross sectional study was conducted in Riyadh, Saudi Arabia, to evaluate the practice, awareness and beliefs of community pharmacists about disposal of unused drugs. Out of 360 subjects who participated in the study, more than 70% returned the unused drugs to the pharmaceutical distributors. Around 80% of the participants confirmed the risk of environmental damage due to the inappropriate disposal of drugs, and 87.5% of them held themselves responsible for preventing such risk. Approximately 85% of surveyed pharmacists believed community pharmacies to be an appropriate location for the collection of unused drugs. There was no significant association between the community pharmacists’ age group and years of practice as community pharmacists with either the awareness of unused medication disposal on environmental hazards, or the beliefs about the appropriate location for collecting unused drugs (*p* > 0.05). The awareness and proactive accountable responsibility, along with community pharmacists’ belief of appointing pharmacies to collect unused drugs, strongly support the institution of drug take-back programs.

## 1. Introduction

In Saudi Arabia, the use of both prescribed and non-prescribed medications continues to increase. The concern for self-medication, regardless of whether they are prescribed items or over-the-counter drugs (OTC), seems to be a serious problem among the public in Saudi Arabia [1]. Such uncontrolled self-medication participates in avoidable excess of drug wastage, which has a negative impact financially and environmentally [2]. The mean drug waste was found to be 25.8% for Saudi families and 41.3% for families from other Gulf countries. A total of $150 million was the estimated expenditure on non-consumed drugs among people living in the Gulf countries [3].

Pharmaceuticals can go into the environment through human and animal excretion as well as through the disposal of unused medications [2,4]. Analgesics, anti-epileptics, beta-blockers and antidepressants were identified in 30 different locations in Sydney Harbor [5]. Additionally, the aforementioned pharmacological classes and other classes such as lipid-lowering agents, estrogens and others were identified on land and in the sea worldwide [6]. The most common sources for environmental contamination of pharmaceuticals included household disposal [7,8,9], industrial waste, hospital influent and effluent and human excreta [4,10]. Additionally, pharmaceuticals were found in low concentrations in surface water, ground water and treated drinking water [11,12,13,14]. Despite their availability in low concentrations in the environment, these pharmaceutical agents affect human health and water wildlife [15,16].

In Saudi Arabia, there is no authorized guideline for the disposal of unused drugs. A feasible option to deal with unused drugs is to return those drugs to the pharmacy [17]. However, this option seems to be seldom done in Saudi Arabia. Based on previous studies, the majority of the Saudi population dispose of their drugs in the household waste, while very few people return them to pharmacies [8,9]. These studies also showed that the awareness of Saudi people about the good and safe practice of drug disposal was fairly low.

Community pharmacists (CPs) have a great potential as accessible healthcare providers, who are approached by customers to recommend a pharmaceutical agent, dispense a prescription and counsel a patient regarding his/her medications. However, CPs’ potential toward drugs disposal directions and practice are not emphasized enough. Therefore, the aim of this study was to evaluate the practice, awareness and beliefs of CPs about the disposal of unused drugs.

## 2. Materials and Methods

A cross sectional survey between July and August 2019 was conducted in the capital of the Kingdom of Saudi Arabia, Riyadh city. The self-administered survey was distributed in-person to investigate the CPs’ practice, awareness and attitude about the disposal of drugs. Based on what about the community pharmacists’ workforce in Saudi Arabia was determined by Alruthia et al. [18], the sample size (with 95% confidence interval and 5% margin of error) was calculated to be 368 participants. A convenient sample of community pharmacies from various areas was selected, based on the geographical areas of Riyadh city.

The questionnaire was adopted from previous studies and aimed to assess the practice and attitude of CPs towards drugs disposal in New Zealand and Kuwait [17,19]. The questionnaire consisted of four sections. The first section included questions about the demographic data of CPs. Three questions, aiming to assess the awareness of CPs towards environmental hazards, were available in the second section. All these questions were assessed utilizing a 5-point Likert scale of 1 to 5. However, the first two questions involved the terms 1 = do not know, 2 = no damage, 3 = no serious damage, 4 = some damage and 5 = serious damage, while the third question involved the terms 1 = strongly disagree, 2 = disagree, 3 = uncertain, 4 = agree and 5 = strongly agree. For suitability of statistical analysis, answers ‘some damage’ and ‘serious damage’ were compound to the response ‘causes damage’, and answers ‘agree’ and ‘strongly agree’ were compound to the answer ‘agree’. The components of the third and fourth section were used to assess the beliefs of CPs associated to future procedures to control unused drugs, and to assess the practice of CPs towards the disposal of unused drugs, respectively. A pilot study was done among five CPs who have at least 5 years of experience working in community pharmacies in Saudi Arabia to assess the validity and reliability of the questionnaire. The content validity using the content validity index with a four-point Likert scale and the reliability using Cronbach’s alpha coefficient were determined to be 0.8 and 0.7, respectively.

All collected data were entered into SPSS version 25, compatible with Windows for analysis. Fisher’s exact test was conducted to find any significant association between respondents’ demographic characteristics and items at a significance level of 0.05.

## 3. Results

The self-administered survey was distributed in-person to 400 CPs, and a total of 360 CPs (with 90% response rate) participated in the study. Table 1 shows the demographics of our participants.

Regardless of the various dosage forms, this survey found that the most common approaches for disposing unused medications were to send the medication back to the pharmaceutical distributor (between 73.3 and 75.3%), followed by putting medication in the medicines’ bin (between 15.5 and 16.7%). Table 2 shows the disposal approaches of unused medications among CPs based on various dosage formulations.

This study assessed the awareness of CPs regarding environmental hazards because of unused disposal drugs (Table 3). About 80% of CPs reported environmental damage as a result of unused medications being thrown in the sink or toilet. A very high percentage of CPs (87.5%) agreed that protecting the environment is one of their individual responsibilities. There was no significant association between the age group and years of practice as community pharmacy of CPs with any of the questions (*p* > 0.05).

For future reclaim programs in Saudi Arabia, the vast majority of the CPs believed that the most appropriate areas to place containers to collect unused medications were inside community pharmacies and pharmacies of public and private hospitals (Table 4). By assessing the impact of years of practice, no significant association was found between the age group of CPs and their years of practice in a community pharmacy with any of the beliefs (*p* > 0.05).

## 4. Discussion

Inappropriate drug disposal can lead to potentially unfavorable consequences. Drug classes, such as antibiotics, analgesics and beta-blockers, have been identified in wastewater [20]. The uncontrolled presence of medications can negatively impact the natural living environment and health. The existence of antimicrobials in the environment may raise concern of antimicrobial resistance. Additionally, possible adverse effects and the unintended exposure risk of particular disposed medications were detected as affecting animals and humans, especially children [21,22,23]. Diclofenac (a non-steroidal anti-inflammatory medication) found in wastewater, for example, was shown to impair the renal function of brown trout [21]. The United States Food & Drug Administration (FDA) warned about fatalities among children due to accidental exposure to fentanyl patches, some of which were inappropriately wasted [22,23].

For this reason, many nations have established policies for the disposal of unused medications. The United States FDA developed governmental guidelines that encourage the availability of disposal instructions on drugs labels, establish a ‘take-back’ program for collecting and disposing unused medications in every city or county, and provide advice on disposing of unwanted drugs appropriately [23]. The integrated efforts of several agencies, such as the Department of Health and Social Care (DHSC), the National Health Services (NHS) of England and NHS Improvement, and the Pharmaceutical Services Negotiating Committee (PSNC) in the United Kingdom, resulted in formulating a policy necessitating community pharmacies to collect the unused medications from the public, and obligating the NHS to make a deal with a medical waste company to collect these medications from pharmacies frequently [24]. The Australian government conducted a similar program named ‘Return Unwanted Medicine’, where unused medications are collected by the community pharmacies [25]. In contrast, there are no policy guidelines for the disposal of unused medications in Saudi Arabia. Additionally, very few studies have been published about the knowledge and practice of appropriate disposal of drugs in the Middle East, particularly in Saudi Arabia [8].

In this study, the majority of CPs (between 73.3 and 75.3%) reported that the pharmaceutical supplier is the main route of disposing of solid or semi-solid as well as liquid unused medications. In contrast to a survey study conducted among pharmacists in Kuwait, about 73% of respondents disposed of unused medications in the trash and the toilet [19]. A similar survey study conducted among CPs in New Zealand revealed that the third-party contractors are a main route of disposal for solid (80%) and semi-solid (61%) unused medications [17]. However, the majority of surveyed CPs tends to dispose of the returned liquid (45%) and scheduled II controlled drugs (58%) into the sink inside the pharmacy. Such practice of disposing of the returned liquid and controlled drugs into sinks, and the practice of burning the unused medications by the third-party contractors, made 90% of community pharmacists in New Zealand call for the need of a destruction system by their health authority.

In Saudi Arabia, the current crucial need is for a policy to be formed by the Ministry of Health (MOH), which would permit community pharmacists to receive returned UMs from the general population and guide the pharmacists on their appropriate disposal [26]. The Saudi MOH published a general guideline for ‘Implementing Regulations of Uniform Law for Medical Waste Management’. This guideline states that each health facility (including pharmacies) should have a contract with a company for waste removal. However, this guideline is general and does not focus on the return medication particularly. In addition, there is no guide or policy for community pharmacies to receive unused medications from the public.

Our CPs showed high awareness (79%) of the inappropriate disposal of medications, which may negatively impact the environment. Moreover, most of them (87.5%) held themselves responsible for protecting the environment from such risks. On the other hand, in another study, around 70% of Saudi drug consumers accounted themselves responsible for the proper way of disposing unused medications, and about 79% of them were willing to receive education or information on the proper way of disposing of unused medications [8]. About 85% of our surveyed pharmacists believe that community pharmacies are an appropriate location for the collection of unused medications. Additionally, the growing Saudi population requires the practice of the community pharmacy to be shifted more toward the paradigm of patient-centered care [27]. All these above-mentioned reasons may require immediate action to establish a drug-take program within community pharmacies in Saudi Arabia.

The study has some limitations. It was conducted only in one region, Riyadh. Saudi Arabia consists of thirteen regions. However, Riyadh is one of the largest regions in terms of population and area [28]. In addition, Riyadh has the highest number of pharmacists that comprise 35.64% of the total pharmacists in Saudi Arabia [18]. Our study was generalized, and doesn’t specify the awareness and beliefs of disposing of ‘hazardous medications’ or ‘controlled-drugs’. However, any drug could carry environmental or human risk if disposed inappropriately. Thus, our objective was to assess the practice, awareness and beliefs of drugs disposal in general regardless of any medications classifications.

## 5. Conclusions

The awareness and proactive accountable responsibility from both pharmacist and society, the beliefs of community pharmacists about the appropriate location for the collection of unused medications and the new practice of the community pharmacy strongly support the institution of drug take-back programs. To launch a national effective drug take-back program, the collaboration of different governmental sectors, along with national awareness on different levels, is necessary.

## Figures and Tables

**Table 1:** Demographics of participating pharmacists in the survey (*n* = 360 *).

Variables		*N* (%)
Age (years)	20–30	175 (48.6)
	31–40	134 (37.2)
	41–50	51 (14.2)
Qualification	B pharma	320 (88.9)
	Pharm D	15 (4.2)
	Master	17 (4.7)
	Diploma	3 (0.8)
Years of practice as community pharmacist	1–4	85 (23.6)
	5–9	156 (43.3)
	More than 10	118 (32.8.2)

* missing data; B pharm: Bachelor degree of pharmaceutical sciences; PharmD: Doctor of pharmacy degree.

**Table 2:** Disposal approaches of unused medications among community pharmacists based on various dosage formulations (*n* = 360 *).

Items	Answers	*N* (%)
SOLID dosage forms	In the rubbish bin	13 (3.6)
	In the sink	4 (1.1)
	In the toilet	7 (1.9)
	In a medicines’ bin	58 (16.1)
	Sent back to pharmaceutical distributor	271 (75.3)
	Other	5 (1.4)
LIQUID dosage forms	In the rubbish bin	6 (1.7)
	In the sink	13 (3.6)
	In the toilet	16 (4.4)
	In a medicines’ bin	55 (15.5)
	Sent back to pharmaceutical distributor	264 (73.3)
	Other	4 (1.1)
SEMI-SOLID preparations	In the rubbish bin	12 (3.3)
	In the sink	5 (1.4)
	In the toilet	10 (2.8)
	In a medicines’ bin	60 (16.7)
	Sent back to pharmaceutical distributor	267 (74.2)
	Other	4 (1.1)

* missing data.

**Table 3:** Awareness among community pharmacists of environmental hazards of unused medication disposal.

Items	Answers	*N* (%)
Effect of drug disposal on the environment	No damage	51 (14.7)
	Damage	284 (78.9)
	I don’t know	23 (6.4)
Damage on the environment if you, as an individual, disposed of unused drugs by throwing them away in the sink or toilet	No damage	51 (14.2)
	Damage	287 (79.7)
	I don’t know	22 (6.1)
Acknowledgment of personal responsibility	Disagree	25 (6.9)
	Uncertain	20 (5.612)
	Agree	315(87.5)

**Table 4:** Beliefs of community pharmacists about the appropriate location for collecting unused drugs.

Items	Answers	*N* (%)
Secure containers inside pharmacies within community pharmacies	Good idea	306 (85.0)
	Uncertain	30 (8.3)
	Not a good idea	23 (6.4)
Secure containers inside pharmacies within government hospitals	Good idea	309 (85.8)
	Uncertain	29 (8.1)
	Not a good idea	21 (5.8)
Secure containers inside pharmacies within private hospitals	Good idea	281 (78.1)
	Uncertain	37 (10.3)
	Not a good idea	41 (11.4)

## Data Availability

Data sharing not applicable.
